# Extracellular vesicles in multiple myeloma: pathogenesis and therapeutic application

**DOI:** 10.1111/febs.70093

**Published:** 2025-04-09

**Authors:** Chloe Wylie, Rebecca Rowan, Dessi Malinova, Lisa Crawford

**Affiliations:** ^1^ Patrick G Johnston Centre for Cancer Research Queen's University Belfast UK; ^2^ Wellcome‐Wolfson Institute for Experimental Medicine Queen's University Belfast UK

**Keywords:** biomarker, bone marrow microenvironment, drug resistance, extracellular vesicles, multiple myeloma

## Abstract

Multiple myeloma (MM), characterised by the clonal proliferation of plasma cells in the bone marrow, is the second most common haematological malignancy worldwide. Although there is now an impressive artillery of therapeutics to tackle this condition, resistance remains a prevalent issue. The bone marrow microenvironment performs a crucial role in supporting MM pathogenesis and promoting the development of therapeutic resistance. Extracellular vesicles (EVs), small vesicles that carry bioactive molecules, are a key component of cell‐to‐cell communication within the bone marrow microenvironment. In this review, we summarise the contribution of EVs to disease progression and anticancer treatment resistance and discuss the potential therapeutic applications of EVs in MM.

## Graphical abstract

Multiple myeloma is characterised by the clonal proliferation of plasma cells in the bone marrow. Extracellular vesicles (EVs) are a key component of cell‐to‐cell communication within the bone marrow microenvironment, promoting tumour growth, bone remodelling, immune modulation, angiogenesis and therapeutic resistance. The clinical application of exosomes is of significant interest both as a potential therapeutic target and as a diagnostic tool.

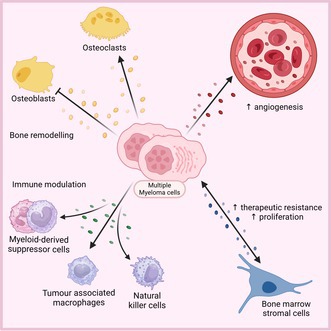

**Abbreviations**
AREG: amphiregulin
BMM: bone marrow microenvironment
BMSC: bone marrow stromal cell
circRNA: circular RNA
DC: dendritic cell
DEX: DC‐derived exosome
ESCRT: endosomal sorting complexes required for transport
EV: extracellular vesicle
lncRNA: long non‐coding RNA
MDSC: myeloid‐derived suppressor cell
MGUS: monoclonal gammopathy of undetermined significance
miRNA: micro RNA
MM: multiple myeloma
OS: overall survival
PFS: progression‐free survival
SM: sphingomyelin
TAMs: tumour‐associated macrophages

## Introduction

Multiple myeloma (MM) is a haematological malignancy characterised by the accumulation of malignant plasma cells in the bone marrow. It accounts for 10–15% of all haematological malignancies, with a worldwide incidence of 176 404 in 2020 [1]. Over the past couple of decades, treatment options for MM have significantly expanded, leading to markedly improved life expectancy. Standard of care therapy typically includes combinations of two or more drugs from a range of classes including proteasome inhibitors (bortezomib, carfilzomib, ixazomib), immunomodulatory drugs (thalidomide, lenalidomide, pomalidomide), monoclonal antibodies (daratumumab, isatuximab), corticosteroids (dexamethasone, prednisone) and alkylating agents (melphalan, cyclophophomide), with or without autologous stem cell transplant [2]. Nevertheless, MM remains largely incurable, with patients becoming refractory to available therapies [3]. This highlights the need to better understand and target resistance mechanisms. MM evolves from a pre‐malignant stage termed monoclonal gammopathy of undetermined significance (MGUS) through to smouldering MM and then to MM. The bone marrow microenvironment (BMM) plays a significant role in the pathogenesis of MM. It consists of stromal cells, immune cells, endothelial cells, mesenchymal stem cells, osteoclasts, osteoblasts and soluble factors which all facilitate MM cell adhesion within the BMM and enable the progression of MM. It is widely accepted that complex interactions between the MM cells and the BMM promote MM cell survival and resistance [4, 5]. A critical mechanism of cellular communication which has received a lot of attention in recent years is trafficking of cargo including proteins and nucleic acids between cells in small membrane‐bound structures termed extracellular vesicles (EVs). This review focuses on the role of EVs in promoting a permissive BMM for MM cell proliferation and survival and explores the potential of EVs for therapeutic applications.

## Extracellular vesicles

**Fig. 1:**
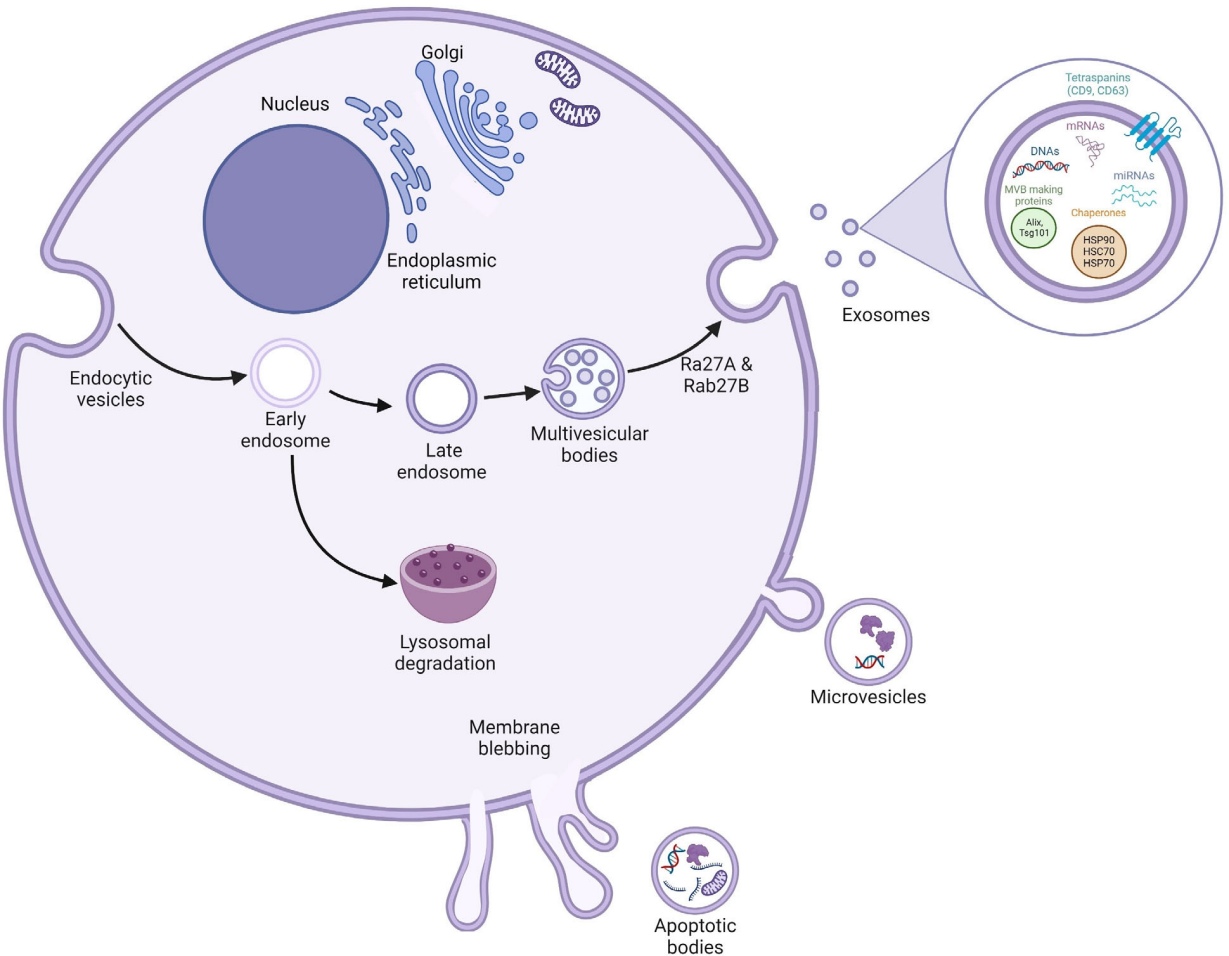
Extracellular vesicles. Extracellular vesicles (exosomes, microvesicles and apoptotic bodies) are lipid‐bound particles that carry bioactive molecules. Microvesicles are produced via the budding and cleavage of the cellular membrane containing components of the cells contents when there is an inward flux of calcium ions into the cell resulting in membrane distortion and eventual vesicle release. Apoptotic bodies are produced when cell death signalling occurs within the parent cell and these vesicles encapsulate cellular contents, enabling more efficient elimination via phagocytes. Endocytic vesicles are formed by the invagination of the plasma membrane which buds off within the cell to form the early endosome, which in turn matures into the late endosome. Inward budding produces multivesicular bodies (MVBs) which are shuttled for release by GTPases Rab27 A/B. At the point the vesicular bodies are released into the extracellular space they are denoted exosomes. Exosomes contain various components such as tetraspanins, mRNA, miRNA, DNA fragments, chaperone proteins and MVB making proteins which are capable of being taken up by recipient cells and altering cellular behaviour and protein production.

Extracellular vesicles are produced by the majority of cell types and carry various molecular cargo that can be delivered to recipient cells. There are three main subtypes of EV: apoptotic bodies, microvesicles and exosomes, as shown in Fig. 1, which are characterised based on differences in size, conditions of production and release from the parent cells, and their biological functions [6]. EVs are considered key players in cell communication, and it is becoming evident that they can influence the pathogenesis of a variety of cancers. Interest and understanding of EVs has grown considerably over the last decade; however, there has not always been consensus on methods to isolate and characterise specific types of EVs, and the International Society for EVs has developed minimal information for studies of EVs (MISEV) guidelines to address this issue [7]. Therefore, although this review focuses predominantly on exosomes, they will be broadly referred to as EVs throughout to encompass the heterogeneity in isolation methods employed across different studies.

### Apoptotic bodies

The largest EVs are apoptotic bodies (50–5000 nm) which are produced by apoptotic cells as a result of membrane blebbing and cell disassembly. Apoptotic bodies compile cellular contents arbitrarily such as DNA fragments, RNA, proteins, lipids and organelles to aid in the elimination of cell debris. These EVs are disposed of via phagocytosis [8]. There is limited knowledge of their biological functions so far, with one study suggesting a role in cellular communication in MM [9].

### Microvesicles

Intermediate in size (100‐1000 nm), microvesicles are formed by pinching off from the plasma membrane. Microvesicle biogenesis is initiated via an influx of intracellular calcium ions which impacts the arrangement of phospholipids in the cell membrane and this distortion causes outward budding of the membrane, enabling these microvesicles to be pinched off into the extracellular space. They are hypothesised to be taken up by recipient cells in an energy‐dependent manner and contain an abundance of protein cargo as well as nucleic acids, lipids and mitochondria [10].

### Exosomes

Exosomes are the smallest EV, typically around 30–200 nm, and are produced by most cell types within the human body. Exosome biogenesis occurs via three sequential stages; endocytic vesicles of the plasma membrane fuse together to produce early endosomes which undergo maturation to late endosomes. Intraluminal vesicles are then produced by late endosomes undergoing inward budding and result in multivesicular bodies [11]. Finally, the multivesicular bodies release exosomes by fusing with the plasma membrane via interaction of Rab27A/Rab27B proteins [12]. Generally, two pathways govern exosome production: (a) the endosomal sorting complexes required for transport (ESCRT) pathway, which is induced via ESCRT complexes [13] or (b) the ESCRT‐independent pathway, which involves molecules such as lipids and tetraphosphates [14, 15, 16]. Once released, exosomes can be taken up by recipient cells in a number of ways: (a) direct interaction of ligands on the surface of exosomes with receptors on the membrane of target cells, (b) membrane fusion whereby exosomes fuse with the plasma membrane of recipient cells to release their content into the cytosol, or (c) through endocytic pathways including phagocytosis, micropinocytosis, lipid‐raft mediated, caveolin‐mediated or clathrin‐mediated endocytosis [17]. Exosomes contain lipids, proteins and RNAs, such as long non‐coding RNAs (lncRNAs), circular RNAs (circRNAs) messenger RNAs (mRNA) and microRNAs (miRNA), encapsulated within a lipid bilayer membrane [18]. In addition to lipids, the exosomal membrane is enriched in tetraspanins, adhesion molecules and transmembrane receptor proteins which facilitate target cell specificity [19]. Exosomes can activate signal transduction on the surface of recipient cells through binding to cell surface receptors and activate intracellular signalling through delivery of cargo. Exosome‐mediated communication has been reported to impact almost all cellular pathways, with the biological influence dependent both on the cell of origin and the target cell. In the context of the tumour microenvironment, exosomes have been shown to alter key signalling pathways including MAPK, PI3K/Akt, mTOR, Wnt, JAK/STAT and T‐cell receptor signalling in an autocrine and paracrine manner to promote tumour growth, survival, migration and immune escape [20, 21, 22, 23, 24, 25].

## Extracellular vesicles in the bone marrow microenvironment

**Fig. 2:**
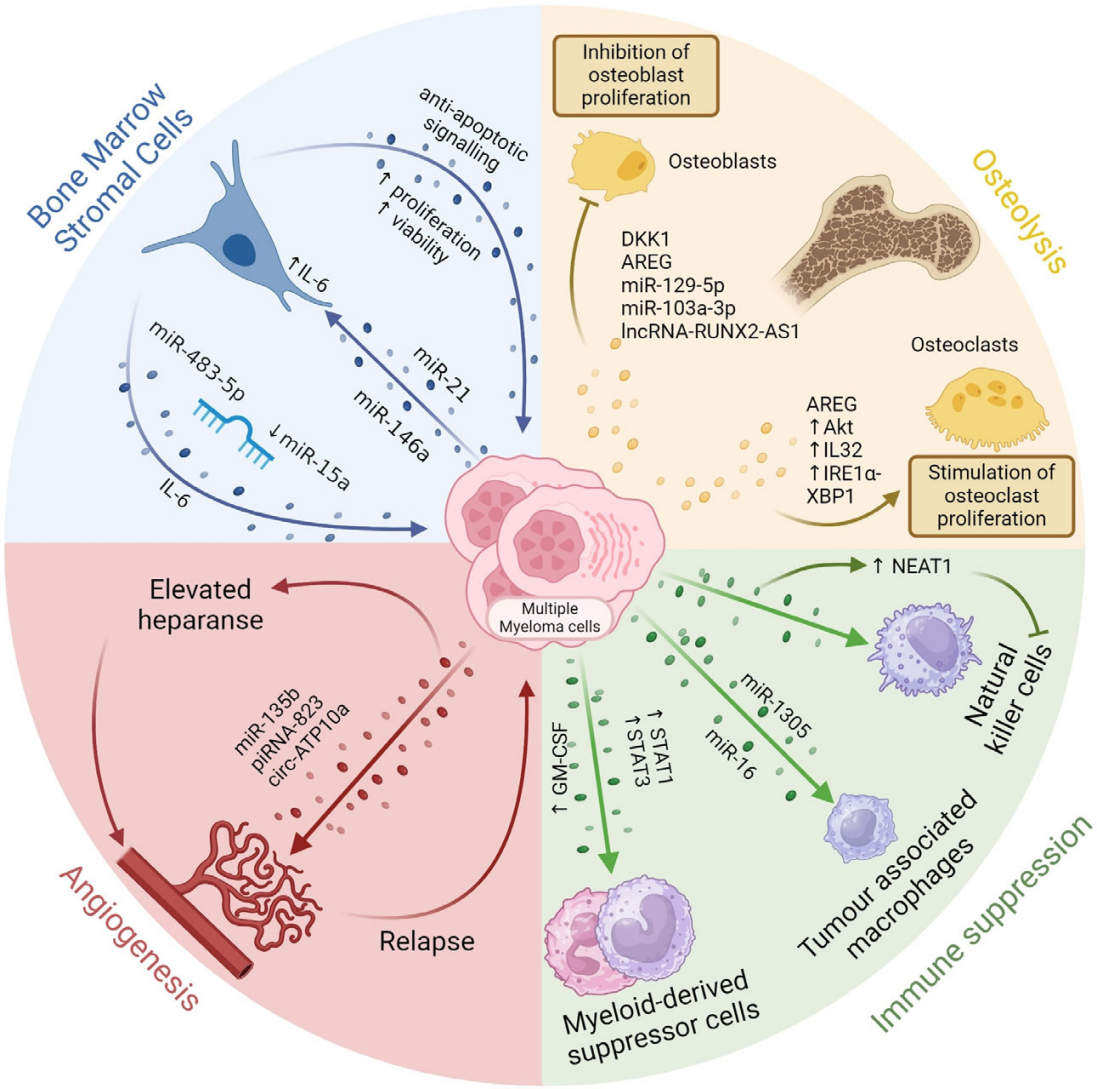
Extracellular vesicles in the bone marrow microenvironment. Extracellular vesicles produced by multiple myeloma cells and bone marrow stromal cells contribute to remodelling of the microenvironment, promoting tumour growth, osteolysis, angiogenesis and immune suppression.

MM cells depend on interactions in the BMM for cell proliferation and survival. EVs are key mediators in the microenvironment promoting tumour growth, angiogenesis, bone remodelling and immune evasion, as summarised below and in Fig. 2.

### Bone marrow stromal cell/MM cell interactions

Bidirectional interactions between MM cells and bone marrow stromal cells (BMSCs) through direct cell‐to‐cell signalling, soluble factors and EVs contribute to tumour growth and resistance to therapy [4, 5]. Functional and phenotypic differences have been described between BMSCs from MM patients compared to healthy donors, and similarly, differences in EV content have also been reported. Roccaro *et al*. [26] demonstrated that EVs derived from MM BMSCs contained less of the tumour suppressor miR15a and a higher level of cytokines than BMSCs from healthy donors, including interleukin‐6 (IL‐6), a key proliferative factor for MM. Complementary to this, EVs from MM cells have been found to transfer miR‐21 and mir‐146a to BMSCs to promote IL‐6 secretion [27, 28]. The miRNA miR‐483‐5p was also reported to be higher in EVs from MM‐derived BMSCs compared to control BMSCs, resulting in increased expression of miR‐483‐5p in MM cells and an associated increase in proliferation [29]. The histone deacetylase (HDAC) inhibitor Panobinostat is approved for MM therapy and has been reported to act on the BMM in addition to tumour cells. Work conducted by Ho *et al*. demonstrated that knockout of HDAC3 in BMSCs interrupted the paracrine signalling loop between BMSCs and MM cells, resulting in decreased MM cell growth. HDAC3 knockout led to a decrease in the production of EVs and a decrease in tumourigenic miRNAs in the EV cargo from these cells [30].

### Angiogenesis

Angiogenesis is a key evolution in the progression of MM that enables tumour growth and dissemination. Increased BM angiogenesis is fuelled predominantly by vascular endothelial growth factor (VEGF) secretion by MM cells and BMSCs and is linked with disease progression [31]. The BM is a naturally hypoxic environment, and studies have reported an increase in the secretion of EVs from hypoxic MM cells. EVs from hypoxic MM cells were enriched for miR‐135b and the enzyme heparanase, both of which contributed to enhanced endothelial tube formation [32, 33]. The piwi‐interacting RNA piRNA‐823 was also found to be enriched in MM cell‐derived EVs (MM‐EVs), particularly those with advanced disease, and promoted the proliferation and invasion of endothelial cells by enhancing the expression of VEGF, IL‐6 and ICAM‐1 [34]. Co‐culture of endothelial cells with BMSC‐EVs, which are abundant in miR‐21, has been shown to promote proliferation, migration and tubular formation [35]. Additionally, circ‐ATP10A was found to be significantly upregulated in EVs derived from patient MM, and this circRNA promoted angiogenesis through sponging of several miRNAs to regulate the expression of key angiogenic signalling molecules VEGFB, HIF1A (hypoxia‐inducible factor 1A), PDGFA (platelet‐derived growth factor A) and the FGF (fibroblast growth factor) family [36]. Interestingly, EVs from bortezomib‐treated MM cells were found to have lower levels of pro‐angiogenic growth factors, including VEGF, than treatment‐naïve cells, resulting in decreased proliferation and migration of endothelial cells [37]. Furthermore, EVs from bortezomib or lenalidomide‐treated cells exhibit reduced expression of VEGF, IL‐6 and FGF, consequently leading to reduced angiogenesis [38].

### Bone remodelling

Osteolytic bone disease is a key feature of MM that occurs due to an imbalance in the activity of osteoblasts, which are bone‐forming cells, and osteoclasts, which promote bone resorption. Crosstalk between MM cells and the BM microenvironment drives increased osteoclast activity and suppression of osteoblast activity to further fuel MM cell growth [39]. Over the past decade, studies have uncovered how MM‐EVs contribute to bone remodelling. Indeed, MM‐EVs have been found to be directly capable of inducing osteolytic lesions in a murine model, highlighting their importance in this process. Faict *et al*. [40] demonstrated that, as well as promoting osteoclast activity, MM‐EVs transferred DKK‐1 to osteoblasts, which blocked the differentiation of the cells and induced apoptosis. Raimondi *et al*. similarly demonstrated that MM‐EVs could act simultaneously on osteoblasts and osteoclasts. Amphiregulin (AREG) was found to be enriched in EVs from MM cell lines and patient samples and promoted osteoclast differentiation through activation of the epidermal growth factor receptor; conversely, AREG blocked osteoblast differentiation [41]. A number of other pathways are known to contribute to EV‐induced promotion of osteoclast activity. For example, MM‐EVs promote the survival of osteoclast precursor cells through upregulation of AKT activity, along with anti‐apoptotic proteins BCL‐XL and survivin, and promote differentiation by inducing the expression of osteoclast markers [42]. MM cells have been shown to secrete the inflammatory cytokine IL‐32 in EVs that induce NFκB signalling in osteoclast precursor cells to promote differentiation and activation of osteoclasts; secretion of IL‐32 by MM cells was further upregulated in response to hypoxia [43]. The unfolded protein response (UPR) has also been implicated in osteoclast differentiation whereby MM‐EVs rapidly activated the IRE1α/XBP1 arm of the UPR, leading to transcription of NFATc1, a pivotal regulator of osteoclast differentiation [44]. On the other hand, non‐coding RNAs from MM‐EVs have been implicated in the repression of osteoblast differentiation. One study found that miR‐129‐5p was enriched in MM‐EVs compared to those from smouldering MM patients and demonstrated that the transfer of miR‐129‐5p to MSCs repressed osteoblastic differentiation through inhibition of the transcription factor Sp1 [45]. Similarly, miR‐103a‐3p from MM‐EVs blocked osteoblastic differentiation [46]. Finally, the transfer of the lncRNA RUNX2‐AS1 from MM EVs to MSCs was found to repress RUNX2 expression, resulting in impaired osteoblastic differentiation [47].

### Immune suppression

Immune dysregulation is a key feature of oncogenesis, and in MM, altered composition of immune cells in the BM microenvironment promotes immune escape and disease progression. Myeloid‐derived suppressor cells (MDSCs), regulatory T cells and tumour‐associated macrophages (TAMs) accumulate in the BMM of MM patients, accompanied by a decrease in cytotoxic T cells [48]. EVs derived both from MM cells and BMSCs from MM patients are taken up by MDSCs and induce MDSC expansion and survival. BMSC‐EVs stimulated STAT1 and STAT3 pathways within MDSCs to amplify BCL‐XL and MCL‐1 expression, fortifying MDSCs pro‐survival response in MM [49]. Similarly, MM‐EVs were found to upregulate STAT3 signalling in MDSCs to promote their growth and viability [50]. EVs from both cell types upregulated nitric oxide release from MDSCs to enhance their suppressive effect on cytotoxic T cells [42, 43]. EVs from MM cells also influence the activity of other immune cell populations. Under hypoxic conditions, MM‐EVs carry increased levels of miR‐1305, which was found to induce polarisation of macrophages into M2‐like TAMs, that in turn can promote tumour progression [51]. MM‐EVs with high levels of miR‐16 have also been reported to induce differentiation of monocytes into M2‐like macrophages, and this effect was increased in EVs from cells with chromosome 13 deletion (del13), a common copy number alteration in MM [52]. Direct uptake of MM‐EVs by T cells of healthy donors was shown to promote apoptosis of CD4+ helper T cells and inhibit the function of cytotoxic CD8+ T cells and regulatory T cells (Tregs) by decreasing secretion of perforin or TGF‐β, respectively [53]. Finally, MM‐EVs have been demonstrated to inhibit natural killer (NK) cell proliferation and activity to further promote MM immune escape through an EZH2/PBX1 axis. MM EVs contain high levels of the lncRNA NEAT1, which acts to downregulate PBX1, a critical transcription factor for NK cell activation, through recruitment of the transcriptional repressor EZH2 [54].

## Extracellular vesicle‐driven resistance mechanisms

While contact‐dependent signalling between MM cells and BMSCs has long been known to confer protection against drug‐induced apoptosis, mounting evidence demonstrates that BMSC‐EVs are also instrumental in mediating resistance mechanisms. Wang *et al*. [55] demonstrated that co‐culture of MM cells with BMSC‐EVs from healthy donors and MM patients significantly abrogated the effect of bortezomib on MM cells by altering the activity of a range of pathways essential in drug resistance, such as Notch 1, Akt and NFκB. Another study found that BMSCs from bortezomib‐resistant MM patients reduced sensitivity of MM cell lines to proteasome inhibitors through EV transfer of the proteasomal subunit PSMA3 and the antisense lncRNA PSMA3‐AS1 [56]. MM cells were also found to selectively uptake miR‐214‐3p and miR‐5100 from EVs secreted by MM patient fibroblasts, enhancing cell survival and promoting resistance to bortezomib through upregulation of MAPK, PI3K and p53 pathways [57]. Additionally, miR‐182 from BMSC‐EVs was found to drive resistance to carfilzomib through negative regulation of the transcription factor SOX6 [58]. EVs derived from bone marrow adipocytes of MM patients induce a protective effect against bortezomib‐induced apoptosis through the transfer of lncRNAs LOC606724 and SNHG1 [59]. Similarly, co‐culture with osteocyte‐EVs reduced the efficacy of a range of MM therapies, including proteasome inhibitors, IMiDs and melphalan, through transfer of miR‐483‐5p and miR‐513a‐5p, which promote a stem‐like phenotype in MM cells [29, 60].

Extracellular vesicles produced from MM cells exposed to therapeutics have been reported to promote resistance in previously untreated MM cells. Exposure to standard of care therapies leads to enhanced secretion of EVs from MM cells. These so‐called chemoexosomes contain altered cargo, in particular high levels of heparanase, leading to remodelling of the extracellular matrix through degradation of heparin sulfate and altered behaviour of cells in the BM microenvironment. Heparanase enhanced ERK signalling and syndecan‐1 shedding in MM cells and stimulated macrophage migration and secretion of TNF‐α to promote chemoresistance [61]. Treatment with melphalan or bortezomib was also reported to induce the secretion of EVs with high levels of acid sphingomyelinase from MM cells, and these EVs were found to abrogate the effect of melphalan or bortezomib in treatment‐naive cells [62]. In another study, Yang *et al*. [63] identified high levels of the lncRNA SNHG16 in EVs from side populations of MM cells, which are cells with a stem‐like phenotype. EVs rich in SNHG16 could confer dexamethasone resistance to the main population of cells through upregulation of the drug resistance markers P‐glycoprotein and multidrug resistance protein 1. Additionally, increased expression of LAMP2 (lysosome‐associated membrane protein 2) which regulates protein loading into EVs, and SORT1 (sortilin 1) which mediates release of EVs, was found to promote secretion of higher levels of EVs from lenalidomide‐resistant MM cell lines. Co‐culture with EVs from resistant cells decreased the efficacy of lenalidomide in matched sensitive cell lines, while conversely silencing of LAMP2 and SORT1 decreased EV secretion and increased sensitivity to lenalidomide [64].

**Fig. 3:**
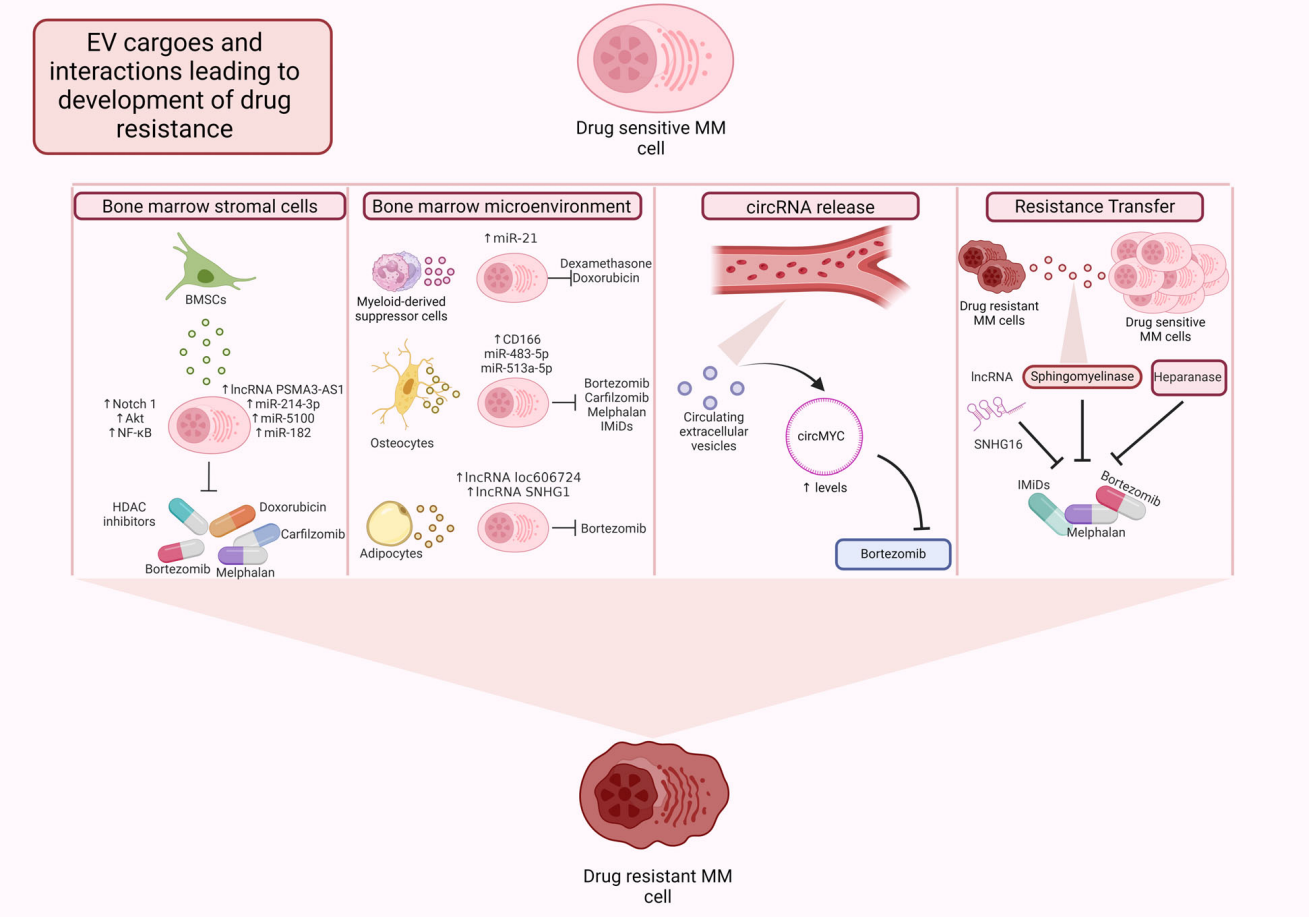
Extracellular vesicle‐driven resistance mechanisms in multiple myeloma.

Studies profiling circulating EVs have identified that EVs from bortezomib‐resistant patients exhibit distinct profiles of lncRNAs, miRNAs and mRNAs which map to signalling pathways commonly associated with therapy resistance, including MTOR and PI3K/AKT [65]. In addition, Luo and Gui [66] found that circulating EVs from bortezomib‐resistant patients exhibited increased levels of circMYC implicating circMYC in drug resistance in MM. Taken together, these studies highlight the important contribution of EV‐mediated communication in promoting drug resistance in MM (Fig. 3) and the critical need to characterise EV content following the development of resistance.

## Circulating EVs as biomarkers of disease progression

There are hundreds of encapsulated cargoes in any given EV and this poses logistical issues in discerning which are critical for exacerbating disease‐specific phenotypes. Extensive databases have been constructed, such as ExoCarta [67], Vesiclepedia [68] and ExMdb [69], with the objective of gaining a deeper understanding of the complex nature of EV cargoes and elucidating disease‐specific biomarkers and molecular targets. Through these and other studies, it is evident that while some cargo is common across EVs from a variety of cell types, other cargo reflects the cell of origin. Recent findings have demonstrated that circulating EVs isolated from peripheral blood contain a unique composition of DNA, RNAs and proteins which may be beneficial as biomarkers of disease progression across a number of malignancies, including MM. One study identified that lower levels of the EV miRNAs let‐7b and miR18a, both of which have been reported to act as tumour suppressors, were predictive of a poorer progression‐free survival (PFS) and overall survival (OS) in MM patients [70]. Another study analysing circulating EV circRNAs from MM patients and healthy individuals reported that elevated levels of circMYC in MM patients correlated with high‐risk cytogenetic markers 17p deletion and t(4;14) and was associated with increased relapse rates and mortality [66]. Proteomic studies revealed a 5‐fold increase in EV protein cargo isolated from peripheral blood, along with elevated production of EV protein cargo in the BM of MM patients, when compared with MGUS patients. No significant difference was observed between MGUS patients and healthy donors [71]. Higher levels of macrophage EV markers CD163 and CD206 have been identified in newly diagnosed MM in comparison to MGUS patients and those with relapsed MM, suggesting that EVs from macrophages may be important in malignant transformation [72]. EVs isolated from MM cell lines and peripheral blood of MM patients were found to be enriched in the transmembrane glycoprotein CD44, which has been implicated in treatment resistance to dexamethasone and lenalidomide. Analysis of MM patients demonstrated that serum CD44 is primarily localised to EVs and increased serum CD44 was associated with a reduced overall survival [73]. The potential of EV miRNAs to predict primary or acquired drug resistance for MM patients was also assessed and identified downregulation of several miRNAs (miR‐16‐5p, miR‐15a‐5p, miR‐20a‐5p and miR‐17‐5p) in EVs from bortezomib‐resistant patients [74]. These studies highlight the potential clinical utility of an EV liquid biopsy to aid diagnosis and disease monitoring in MM.

## Therapeutic applications of EVs in MM

Extracellular vesicles as key communicators in the MM tumour microenvironment offer a variety of potential avenues for therapeutic intervention, such as targeting EV secretion, targeting aberrant miRNAs and manipulating EV cargo. There are numerous regulators in the formation and release of EVs representing targets which can be pharmacologically disrupted to prevent EV release [75]. The most widely used compound to block EV biogenesis and release is the neutral sphingomyelinase (SMase) inhibitor GW4869. SMase catalyses the breakdown of sphingomyelin (SM) into ceramide; therefore, through inhibition of SMase, GW4869 blocks the ceramide‐dependent budding and release of exosomes [76]. Two individual studies reported that inhibition of exosome secretion using GW4869 reduced osteolysis in *in vivo* models of MM [40, 77] and furthermore, Faict *et al*. [40] found that GW4869 enhanced response to bortezomib. The GTPase RAB27A, which aids in shuttling EVs to the plasma cell membrane for release, can also be targeted [12]. Two compounds, nexinhib4 and nexinhib20, were identified to inhibit the interaction between RAB27A and JFC1 and therefore prevent exocytosis of intracellular vesicles [78]. The farnesyl transferase inhibitor tipifarnib has also been reported to decrease exosome release by downregulating the expression of RAB27A [79]. Although not explored in the context of EV release, tipifarnib has been shown to enhance the effect of bortezomib in MM [80]. Several other compounds targeting proteins across the various stages of exosome biogenesis and release have been explored in other cancer types and are extensively reviewed in [75].

The abnormal expression of EV cargo in MM can also act as a potential therapeutic target. For example, heparanase, which is abundant in MM‐EVs following treatment, promotes resistance in MM cells and also regulates EV formation and secretion [61, 81]. The heparanase inhibitor Roneparstat was found to sensitise MM cells to standard of care therapies and reduce tumour relapse [82, 83]. In addition, inhibition of miR‐21, which is enriched in EVs from MM BMSCs, blocks the EV‐induced promotion of angiogenesis and can also inhibit MM cell growth [35, 84].

Extracellular vesicles have been recognised for their promise as drug delivery vehicles and for use as cancer immunotherapy due to their size, low immunogenicity, and good stability [85, 86]. For example, in pre‐clinical studies, MM‐EVs have been harnessed to stimulate anti‐tumour immunity. The MM cell line J558 was engineered to express membrane‐bound heat shock protein 70 (HSP70), a molecular chaperone that can activate an anti‐tumour immune response. EVs purified from these cells efficiently stimulated dendritic cell maturation and enhanced CD8+ cytotoxic T‐cell responses. Vaccination with EVs from J558 HSP70 overexpressing cells significantly delayed tumour growth in mice subsequently injected with J558 MM cells, demonstrating the potential of EVs to induce anti‐tumour immunity [87]. Dendritic cells (DCs) play an important role in activating tumour immune responses, and DC‐derived exosomes (DEX) represent another promising immunotherapeutic approach; although not yet explored for MM, early clinical trials using DEX have been found to be a safe and feasible anticancer therapy [88]. Another exciting approach is the use of EVs as an alternative to bispecific antibodies. Xu *et al*. [89] genetically engineered EVs to express CD3 and CD38 antibodies on their surface, allowing them to bind T cells and MM cells, respectively, thereby activating T cells to specifically target MM cells. Another study engineered monocyte‐derived EVs to express anti‐B cell maturation antigen (BCMA), a protein highly expressed on the surface of MM cells, and loaded the EVs with bortezomib by electroporation. These EVs efficiently delivered bortezomib to MM cells *in vivo* and have the potential to overcome dose‐limiting side effects [90]. Additionally, EV‐based bispecifics could be loaded with anti‐MM therapy for more effective tumour elimination.

## Conclusion and future perspective

Although a heavy artillery of combination treatments has been established for MM over the last number of decades, resistance remains a major clinical hurdle. It is widely accepted that the interplay between components of the BMM and malignant plasma cells enables progression and therapy resistance in MM. In recent years, a key role of EVs secreted by cells in the BMM has been uncovered, along with opportunities for therapeutic intervention. MM cells have been reported to secrete increased levels of EVs compared to healthy counterparts, and therefore blocking EV biogenesis and secretion presents one potential therapeutic strategy. There are a number of EV inhibitors available, including clinical stage compounds like Tipifarnib, a farnesyltransferase inhibitor that demonstrates an inhibitory effect on exosome release; however, more selective inhibitors would be advantageous for future drug development. Targeted therapeutic options arise from the potential to target specific EV cargo that contribute to disease progression, immune evasion, or resistance. Larger studies characterising EV cargo from genetic subtypes and relapse patients will help to advance this field. Finally, a key area for future development lies in harnessing the potential of EVs as an immunotherapy. Over the few years, immunotherapies such as CAR‐T, antibody‐drug conjugates and bispecific T‐cell engagers have entered the clinic for MM and demonstrate clinical efficacy in relapsed patients. Further studies to optimise and standardise large‐scale preparation of EVs will help to realise their clinical application as an anti‐MM immunotherapy.

## Conflict of interest

The authors declare no conflict of interest.

## Author contributions

CW, RR, DM and LC wrote the manuscript; CW created the figures.
